# Low usage of government healthcare facilities for acute respiratory infections in guatemala: implications for influenza surveillance

**DOI:** 10.1186/1471-2458-11-885

**Published:** 2011-11-24

**Authors:** Kim A Lindblade, April J Johnson, Wences Arvelo, Xingyou Zhang, Hannah T Jordan, Lissette Reyes, Alicia M Fry, Norma Padilla

**Affiliations:** 1 Centers for Disease Control and Prevention (CDC), 1600 Clifton Rd., Atlanta GA, 30333 USA; 2 CDC Regional Office for Central America and Panama, 18 Avenida, 11-95 Zona 15, Guatemala City, Guatemala; 3 Epidemic Intelligence Service, CDC, Atlanta, GA 30333 USA; 4 Field Epidemiology Training Program, Ministerio de Salud Pública y Asistencia Social, 6 Avenida 3-45 Zona 11, Guatemala City, Guatemala; 5 CDC-UVG Collaboration, Universidad del Valle de Guatemala,18 Avenida, 11-95 Zona 15, Guatemala City, Guatemala

## Abstract

**Background:**

Sentinel surveillance for severe acute respiratory infections in hospitals and influenza-like illness in ambulatory clinics is recommended to assist in global pandemic influenza preparedness. Healthcare utilization patterns will affect the generalizability of data from sentinel sites and the potential to use them to estimate burden of disease. The objective of this study was to measure healthcare utilization patterns in Guatemala to inform the establishment of a sentinel surveillance system for influenza and other respiratory infections, and allow estimation of disease burden.

**Methods:**

We used a stratified, two-stage cluster survey sample to select 1200 households from the Department of Santa Rosa. Trained interviewers screened household residents for self-reported pneumonia in the last year and influenza-like illness (ILI) in the last month and asked about healthcare utilization for each illness episode.

**Results:**

We surveyed 1131 (94%) households and 5449 residents between October and December 2006 and identified 323 (6%) cases of pneumonia and 628 (13%) cases of ILI. Treatment for pneumonia outside the home was sought by 92% of the children <5 years old and 73% of the persons aged five years and older. For both children <5 years old (53%) and persons aged five years and older (31%) who reported pneumonia, private clinics were the most frequently reported source of care. For ILI, treatment was sought outside the home by 81% of children <5 years old and 65% of persons aged five years and older. Government ambulatory clinics were the most frequently sought source of care for ILI both for children <5 years old (41%) and persons aged five years and older (36%).

**Conclusions:**

Sentinel surveillance for influenza and other respiratory infections based in government health facilities in Guatemala will significantly underestimate the burden of disease. Adjustment for healthcare utilization practices will permit more accurate estimation of the incidence of influenza and other respiratory pathogens in the community.

## Background

As the 2009 influenza A (H1N1) pandemic highlighted, surveillance for influenza is now a worldwide priority.[1,2] At the 58^th ^World Assembly in 2005, The World Health Organization adopted a resolution calling for Member States to fortify and coordinate national strategies to prepare for an influenza pandemic, including establishment of surveillance systems for human influenza.[3] To assist with the development of standardized influenza surveillance systems in the Americas, the Pan American Health Organization (PAHO) and the United States Centers for Disease Control and Prevention (CDC) developed a generic protocol for influenza surveillance incorporating two sentinel surveillance systems, one hospital-based system for severe acute respiratory infections (SARI) and SARI-related mortality and another for influenza-like illness (ILI) based in ambulatory clinics.[4]

Sentinel surveillance for influenza can provide information on trends in viral circulation patterns and seasonality, along with virus characteristics to help guide decisions on vaccine composition. However, healthcare seeking behaviors can affect who accesses care at the sentinel site, limiting the ability to gather information to guide public health policies. Without understanding patterns of healthcare seeking behavior, it is not possible to calculate the burden of disease, generalize findings to a larger population or identify risk groups.

Healthcare utilization surveys (HUS), one method of determining the healthcare utilization practices for specific diseases in defined populations, have been conducted in several countries. [5-12] In HUS, random samples of the catchment population are interviewed with respect to their healthcare seeking and treatment behaviors during recent episodes of disease. These data can be used in a number of ways to support the interpretation of information from sentinel surveillance sites: first, to establish correction factors for estimates of incidence in the community based on numbers of cases presenting at the sentinel surveillance site, including the incidence in particular population sub-groups; second, to describe the actual catchment population accessing healthcare at the sentinel site to determine generalizability; and third, to identify other healthcare providers who may be recruited to participate in the surveillance system.

To inform the establishment of a surveillance system for influenza and other respiratory infections, and the implementation of the PAHO/CDC standard protocol for influenza surveillance in Guatemala, we conducted a HUS among residents of the Department of Santa Rosa, Guatemala to describe the healthcare seeking behavior for acute respiratory illnesses.

## Methods

### Study area and population

Guatemala, with a population of 12,755,366 in 2008, had a gross national income per capita of $2680 and is considered a middle-income country by the World Bank (http://data.worldbank.org/indicator/NY.GNP.PCAP.CD, accessed on 1 September 2010). Guatemala is divided into 22 departments, which are further subdivided into 10-29 *municipios *(similar to counties), made up of multiple communities. The Guatemalan Ministry of Public Health and Social Welfare (MSPAS) provides free healthcare in several different settings, including hospitals, health centers, health posts, and outreach centers. Hospitals and health centers are staffed by physicians and nurses, whereas health posts are staffed by nurses. Outreach centers provide preventive and primary healthcare but are only visited by trained medical staff a few days each month. In addition to the MSPAS facilities, formally-employed workers who contribute to the Guatemalan Institute of Social Security (IGSS) can receive healthcare from IGSS hospitals and health centers, which are concentrated in Guatemala City. Other non-governmental locations where people may seek healthcare are private hospitals and clinics, pharmacies, drug shops, and traditional healers and midwives. Communities in Guatemala vary in their access to healthcare depending on their size and location (e.g., urban vs. rural).

Santa Rosa is a mostly agrarian department located in the southeastern part of the country approximately 80 km from Guatemala City. The population in 2006 was 308,522 residing in 14 *municipios *with an estimated 768 communities. Cuilapa is the department's capital city. In contrast to the country as a whole, which is almost half Amerindian indigenous, only 3% of Santa Rosa's residents are Mayan or Xinca, and Spanish is spoken by approximately 91% of the inhabitants. The mortality rate of children <5 years of age for Santa Rosa is 58 per 1000 live births, significantly higher than the average for the country (45 per 1000 live births).[13]

Government-run healthcare facilities within the department include one hospital (the National Hospital of Cuilapa, 176 beds), 14 health centers (one in each *municipio*) and 56 health posts in the outlying communities. There is one IGGS facility that treats only patients involved in motor vehicle accidents. Two small private hospitals as well as approximately 133 private ambulatory clinics are available for those who choose to pay for healthcare. Additionally, healthcare services can be sought from more than 114 pharmacies or drug shops, and an unknown number of traditional healers, midwives and community healthcare workers. Healthcare may also be accessed in Guatemala City and neighboring departments.

### Study design and sampling methods

We conducted a cross-sectional survey to determine healthcare utilization patterns for acute respiratory, diarrhea, neurologic and febrile illnesses: we report here only the results for acute respiratory infections. We used a stratified, two-stage cluster sampling procedure. Communities in the 2002 Guatemalan census were stratified as to whether or not they had a hospital or health center located in their community. As the first stage of sampling, 30 communities were selected within each stratum, using probability proportional to the population of each community, for a total of 60 clusters. Maps detailing household locations were obtained from the Guatemalan Census Bureau for these communities and 20 houses were randomly selected for a total of 1200 houses.

Interviews were conducted in person from October 1 through December 13, 2006. All persons who had lived in the house for at least six of the preceding 12 months were considered members of the household and eligible for inclusion, including persons deceased at the time of the survey if they had been resident during the reference period. Infants <6 months of age were included if they had lived in the household since birth. Households were excluded if a head of household or consenting adult was unavailable after visiting the house on three separate occasions over at least two days, or if the household head declined to participate. Excluded households were not replaced. If a house was abandoned or no longer existed, another house was randomly chosen for inclusion.

### Human subjects

The adult respondents from each household were read a consent statement and asked to give verbal consent for their household's participation. The protocol for this study was reviewed and approved by the institutional review boards of the Centers for Disease Control and Prevention (Atlanta, GA) and the Universidad del Valle de Guatemala (Guatemala City, Guatemala) and approved by the MSPAS (Guatemala City, Guatemala).

### Sample size

The sample size of 600 households per strata (1200 total) was based on calculations for diarrhea, a more common syndrome, rather than for pneumonia as information was not available on the expected incidence of pneumonia. However, assuming a 10% household non-response rate, and 4.8 persons per household, a sample of 600 households per stratum should yield between 39 and 156 persons with pneumonia, assuming an annual incidence of between 1.5% and 6.0%, respectively. Given a design effect of two due to the clustering of pneumonia cases by community and household, this sample would be large enough to estimate the proportion of the population seeking healthcare outside of the house for pneumonia with a precision of 10%, assuming 70% of persons with pneumonia seek care for their illness.

### Survey instrument

A structured survey was completed for each participating household with information on both household- and individual-level characteristics. All household members were enumerated and an adult proxy was interviewed for children <15 years old or older residents not present at the time of the interview to determine whether any household member met any of the case definitions (mild or severe respiratory, diarrhea, acute febrile and acute neurologic illness) during the prescribed time period. A clinical history was obtained for each illness episode (if more than two episodes of the same illness were reported, the most recent illness episode was used as the reference) along with a history of healthcare treatment seeking. Proxies of household residents who died but met the case definition for one of the illnesses in the relevant time period before death were also administered the illness-specific forms.

### Definitions

A case of severe respiratory illness, referred to as pneumonia, was defined as self-reported cough and difficulty breathing for two or more days, or a physician-diagnosis of pneumonia; the reference period was the 12 months prior to the interview. This case definition has been used in several studies of self-reported pneumonia in the community[12,14] and is based on questions that were moderately sensitive and specific for pneumonia from a World Health Organization verbal autopsy questionnaire.[15] Additionally, severe pneumonia was defined for children <3 years old who met the pneumonia case definition as any of the following: blue lips and/or nails, inability to breastfeed or drink, convulsions, unconsciousness or decreased activity. For those ≥3 years old who met the pneumonia case definition, severe pneumonia was defined as fast breathing with confusion.

A case of mild respiratory illness, referred to as ILI, was defined as subjective fever with either cough or sore throat in the 30 days prior to the interview. If a respondent reported both an ILI and pneumonia for the same month, the ILI data were excluded.

Outpatient providers were defined as all sources of healthcare that did not admit patients for overnight stays, and included government and private ambulatory clinics, pharmacies, drug shops, the IGSS and traditional healers. Inpatient providers included government and private hospitals.

### Data management and analysis

Survey forms were received at the offices of the CDC-UVG Collaboration at the Universidad del Valle de Guatemala for optical scanning into a database using the Cardiff Teleform system (Vista, CA). Each Teleform entry was checked manually with the original forms to ensure accurate scanning and coding. Socioeconomic status (SES) was estimated using a wealth index generated using the factor effects derived from the first principle component of a principle component analysis of household goods, house construction material, source of water supply, source of cooking fuel and sanitation facility; the wealth index was categorized into quintiles with households weighted by number of residents and sample weights.[16,17] To account for the complex survey design, sample weights were applied in all analyses. We used the Wald Chi-square statistic to test for differences between proportions and logistic regression to test for trends related to age and SES. Analyses were conducted with SAS version 9.1 (SAS Institute, Cary, NC) using PROC SURVEYFREQ or PROC SURVEYLOGISTIC.

## Results

We approached 1200 households but residents could not be reached at 33 (3%) locations after three visits, and in 36 (3%), the household head declined to participate. We interviewed residents from 1131 (94%) households and gathered information on a total of 5449 persons of which 2806 (52%) were female and 586 (12%) were children <5 years old (Table 1).

**Table 1:** Sociodemographic characteristics among the population surveyed, and those with pneumonia in the previous 12 months or influenza-like illness (ILI) in the previous month, Santa Rosa, Guatemala, 2006^a^

	Sample N = 5449 n (%)	Pneumonia Cases N = 323 n (%)	*P *value	ILI Cases N = 628 n (%)	*P *value ^b^
Sex, female	2844 (52)	165 (50)	0.51	344 (53)	0.44
					
Age (years)			0.76		0.01
0-4	586 (12)	60 (20)		106 (17)	
5-14	1484 (28)	68 (21)		170 (29)	
15-29	1466 (27)	68 (21)		147 (22)	
30-59	1439 (25)	87 (25)		150 (24)	
≥60	474 (8)	40 (12)		55 (8)	
					
Wealth index					
Poorest	832 (21)	49 (20)	0.14	127 (27)	<0.001
Second	927 (21)	51 (20)		127 (25)	
Third	1006 (20)	61 (21)		113 (18)	
Fourth	1141 (19)	84 (23)		127 (17)	
Wealthiest	1543 (19)	78 (15)		134 (14)	

^a ^Percentages are calculated using sample weights.

^b ^*P *values compare characteristics of pneumonia (or ILI) cases with those persons without pneumonia (or ILI).

We found 323 persons (6%, 95% confidence interval [CI] 6-7%) who met the pneumonia case definition in the previous year. Almost all (87%) met the case definition with self-reported cough and difficulty breathing for at least two days; 2% reported only a physician's diagnosis of pneumonia; and 12% reported both. There were 60 cases (11%, 95% CI 9-13%) of pneumonia reported among children <5 years old, and 263 cases (6%, 95% CI 5-6%) among persons aged five years or older. Among the children <5 years old, 31 (6%, 95% CI 5-7%) met the case definition for severe pneumonia.

**Figure 1:**
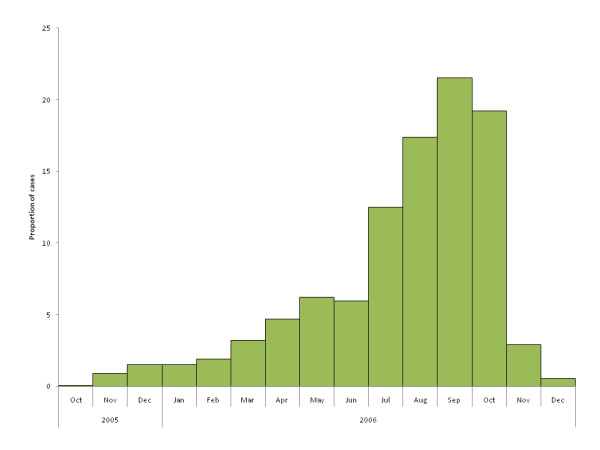
**Proportion of pneumonia cases reported by month of illness onset, Santa Rosa, Guatemala, October-December 2006**.

The proportion of pneumonia cases reported by month increased from October 2005 through September 2006 (Figure 1.) More than half of the pneumonia cases were reported from the last five months prior to the survey.

There were 628 (13%, 95% CI 12-14%) persons who reported ILI in the previous month. A case of ILI was reported by 106 (19%, 95% CI 17-20%) children <5 years old and 522 (12%, 95 CI 11-13%) persons aged five years or older.

### Characteristics of respondents with pneumonia and ILI

The most common symptoms reported by persons with pneumonia were difficult breathing (100%), cough (99%), and feverishness (88%) (Table 2). The mean duration of illness of all persons with pneumonia was 13 days (range 2-120); more than one-quarter had symptoms for seven days or more.

**Table 2:** Frequency of symptoms in people reporting pneumonia in the previous year and influenza-like illness (ILI) in the previous month, Santa Rosa, Guatemala, 2006^a,^^b^

	Pneumonia N = 323 n (%)	ILI N = 628 n (%)
Difficult or fast breathing	322 (100)	309 (51)
Cough	320 (99)	551 (88)
Feverishness	277 (88)	628 (100)
Chills	213 (77)	457 (77)
Wheezing	214 (69)	246 (42)
Confusion or delirium	33 (12)	NA
Hemoptysis	20 (7)	NA
Unconsciousness	23 (7)	NA
Signs of severe pneumonia ^c^	58 (19)	NA
Sore throat	NA	605 (98)
Headache	NA	533 (89)
Muscle aches	NA	450 (76)
Runny nose	NA	447 (71)
Decreased activity	NA	363 (60)
Nausea or vomiting	NA	154 (24)
Duration of illness ≥7 days	91 (29)	353 (56)

^a ^NA = Not applicable

^b ^Percentages are a proportion of non-missing data and are calculated using sample weights.

^c ^Severe pneumonia: children <3 years old: blue lips and/or nails, inability to breastfeed or drink, convulsions, unconsciousness or decreased activity; persons ≥3 years old: fast breathing with confusion.

Among persons who reported ILI, the most common symptom besides feverishness (100%) was sore throat (97%), headache (89%) and cough (88%). Signs of lower respiratory tract infection, such as difficult or fast breathing and wheezing, were less common among person who reported ILI than those with pneumonia. The mean duration of illness among persons with ILI was seven days (range 1-90); more than half of person who reported an ILI had symptoms for seven days or more.

The age distribution of persons with pneumonia was significantly different from the surveyed population without pneumonia (*P*<0.0001), with more children <5 years and adults ≥60 years old among the persons reporting pneumonia than among the surveyed population without pneumonia (Table 1). Similarly, the age distribution of those with ILI was younger than the surveyed population without ILI (*P *= 0.001). The distribution of the person who reported ILI by household wealth index was significantly different from those without ILI (*P*<0.0001) with more cases among persons in the lowest wealth category and fewer in the wealthiest category. There was no difference in household wealth between persons with and without pneumonia (*P = *0.14).

### Healthcare-seeking behavior

Among the 60 children <5 years old reporting pneumonia in the last year, 55 (92%) sought care outside the home. All subsequent analyses of healthcare-seeking behavior are based on those who sought care outside the home. Sixteen (27%) children sought care from more than one source. Hospitals were consulted by 17 (25%) children <5 years old with pneumonia, and most were government hospitals (Table 3). Nine (12%) children <5 years old with pneumonia were admitted for at least one night in a hospital. Outpatient care providers were visited by 38 (75%) children <5 years old with reported pneumonia. Overall, the most frequently reported source of healthcare for children <5 years old with pneumonia were private ambulatory clinics, which attended to more than half the reported cases. More than half (55%) of the children <5 years old with reported pneumonia received care at least once during their illness from government facilities, either government hospitals or ambulatory clinics.

**Table 3:** Healthcare providers consulted by respondents who sought care outside the home for pneumonia in the last year or influenza-like illness in the last month, Santa Rosa, Guatemala, 2006^a^

	Children <5 years old	Persons five years and older
Source of healthcare	n (%)	n (%)
Pneumonia	(N = 55)	(N = 199)
Hospital	17 (25)	28 (12)
Government hospital	16 (24)	22 (11)
Private hospital	2 (1)	6 (1)
Outpatient providers	38 (75)	171 (88)
Government clinic	17 (31)	46 (27)
Private clinic	26 (53)	65 (31)
Pharmacy	4 (6)	45 (16)
Traditional healer	2 (3)	11 (7)
Drug shop	3 (7)	13 (8)
Social Security clinic	1 (3)	3 (2)
Other	0 (0)	10 (7)
Influenza-like illness	(N = 87)	(N = 337)
Hospital	6 (5)	3 (0.2)
Government hospital	5 (5)	2 (0.1)
Private hospital	1 (0.3)	1 (0.1)
Outpatient providers	81 (95)	334 (100)
Government clinic	34 (41)	111 (36)
Private clinic	20 (20)	53 (13)
Pharmacy	12 (13)	110 (29)
Traditional healer	4 (3)	4 (2)
Drug shop	12 (17)	50 (17)
Social Security clinic	1 (1)	0 (0)
Other	3 (3)	17 (5)

^a^Numbers will not necessarily add up to 100% because more than one healthcare provider can be consulted in the course of an illness. Percentages are calculated using sample weights.

Among the 263 persons five years or older who reported pneumonia in the last year, 199 (73%) sought healthcare outside their home, with 21 (8%) seeking care from more than one source. Among persons in this age group who sought care outside the home, 28 (12%) sought care at hospitals (Table 3), and 8 (4%) were admitted for at least one night. Government hospitals provided most of the hospitalized care. Among outpatient care providers, the most frequently sought source of care were private clinics, which provided care to 65 (31%) persons with pneumonia aged five years or older, along with government ambulatory clinics (46, 27%). A considerable proportion (16%) of persons with pneumonia aged five years or older sought care at pharmacies.

Care for ILI was sought outside the home by 87 (81%) children <5 years old, and 6 (6%) sought care from multiple sources (Table 3). Government clinics were the source of healthcare most often consulted by children <5 years old for ILI; 34 (41%) children <5 years old reported seeking care at a government clinic, whereas 20 (20%) reported consulting a private clinic. Hospitals were consulted by 6 (5%) children <5 years old for ILI, and 4 (5%) were hospitalized for one night or more. Care was sought at pharmacies and drug shops for nearly one-third of children <5 years old with ILI.

Care for ILI was sought outside the home by 337 (65%) persons aged five years or older, and 13 (3%) consulted multiple sources. Government clinics were consulted for ILI by 111 (36%) persons aged five years or older. Pharmacies were consulted for ILI by 110 (29%) persons aged five years or older. One (0.1%) ILI patient five years or older was hospitalized for more than one night.

Among the respondents with pneumonia who did not seek healthcare for their illness, the perception that their illness was not severe enough to warrant treatment (28/69, 42%) and the cost of treatment (13/69, 20%) were the major reasons cited for not seeking care. Among persons with ILI who did not seek healthcare for their illness, insufficient severity of illness (68/204, 31%), cost of treatment (37, 18%), lack of medical services (13, 9%) and spontaneous improvement (19, 7%) were the major reasons cited.

### Sociodemographic and illness characteristics associated with seeking treatment at government facilities

There was no significant association between sex of the respondent and whether care was sought for pneumonia (*P *= 0.34) or ILI (*P = *0.15) at a government hospital or clinic (Table 4). Children <5 years old were more likely to receive healthcare for pneumonia and ILI at government facilities than persons aged five years or older, but this difference was statistically significant only for pneumonia (*P = *0.03). There was a significant inverse trend across SES status with persons of higher socioeconomic status less likely to seek care for pneumonia and ILI at government facilities (*P = *0.005 and *P = *0.001, respectively). The duration of illness was not associated with consultation at a government facility for either pneumonia (*P = *0.37) or ILI (*P = *0.25). Severity of pneumonia was not associated with seeking care from a government facility (*P = *0.13).

**Table 4:** Association between sociodemographic and illness characteristics and consultation for healthcare at government facilities for pneumonia or influenza-like illness (ILI), Santa Rosa, Guatemala, 2006

	Pneumonia cases			ILI cases		
	N = 254			N = 424		
	Public	Private		Public	Private	
Characteristic	n (%)	n (%)	*P *value	n (%)	n (%)	*P *value
Sex, female	54 (22)	83 (32)	0.34			0.15
Age (years)			0.0009			<0.0001
<5	29 (29)	26 (21)		38 (24)	49 (18)	
5-14	25 (31)	28 (17)		53 (26)	56 (22)	
15-29	16 (16)	34 (22)		24 (16)	66 (21)	
30-59	16 (14)	43 (23)		28 (20)	75 (28)	
≥60	10 (10)	27 (17)		8 (4)	27 (10)	
Wealth index			<0.0001			<0.0001
Poorest	20 (26)	15 (14)		46 (37)	38 (19)	
Second	18 (22)	17 (14)		30 (24)	61 (30)	
Third	16 (18)	34 (25)		35 (19)	41 (16)	
Fourth	24 (21)	46 (28)		26 (16)	52 (15)	
Wealthiest	18 (12)	46 (18)		14 (4)	81 (20)	
Signs of severe pneumonia	26 (27)	29 (21)	0.08	NA	NA	
Duration of illness <7 days	22 (26)	40 (23)	0.37	93 (60)	147 (56)	0.25

^a^NA = not applicable; percentages are calculated using sample weights.

## Discussion

We conducted a HUS to help inform establishment of a sentinel surveillance system for pneumonia and influenza-like illness in Santa Rosa, Guatemala, and found that private clinics are the single most important source of healthcare for pneumonia both in children <5 years old and older persons. For more mild ILI, both children <5 years old and persons aged five years or older are more likely to consult government clinics than other sources of health care. These findings suggest that in Santa Rosa, sentinel surveillance for pneumonia in government hospitals will significantly underestimate the burden of disease, by up to 75% for children <5 years old and 88% for persons aged five years and older. Government healthcare clinics will underestimate the number of cases of ILI by about 59% for children <5 years old and 64% for persons five years and older.

Our study is consistent with other studies of healthcare-seeking behavior in Guatemala and Central America that have found private clinics to be common sources of healthcare. Van der Stufyt *et al*. reported that more than 40% of Guatemalan families sought healthcare for their children <5 years old from private physicians, compared with 26% consulting a governmental health center.[18] Focus group interviews from three countries in Central America found that persons considered the healthcare obtained through private physicians and clinics preferable to public options because of prompt attention and a perception that healthcare is better.[19] Another study evaluating healthcare utilization among children in rural Guatemala who reported a diarrheal or respiratory illness found private physicians were more likely to be consulted by households with higher income.[20]

The population that uses government health clinics and hospitals for respiratory illnesses in Santa Rosa is younger and poorer than the general population. We found a trend for decreasing use of government facilities with increasing age and household wealth. These findings should be taken into account to improve the generalizability of burden of disease estimates made from sentinel surveillance data.

The results of this study are subject to several important limitations. We used a case definition that required self-report or report by a proxy of an illness that occurred up to one year prior to interview. It is well known that such self-reports are limited by recall decay, and recent episodes are more likely to be recalled than earlier episodes.[21] This could be noted in our data that demonstrated a decreasing report of pneumonia with increasing time before the survey. As long as recent illness episodes do not differ from prior episodes with regard to patterns of healthcare-seeking behaviors, there is no reason to believe that recall decay causes bias with regard to these variables. Because our case definition is based on self-report, there is substantial potential for misclassification, especially between mild and severe acute respiratory illness, and this can be seen in the report of some lower respiratory tract symptoms among respondents with ILI. However, the behaviors associated with episodes of ILI compared to pneumonia (lower probability of seeking care outside the home, less likely to seek treatment at a hospital) are suggestive of a more mild illness and we are reasonably confident that we have described the healthcare seeking behaviors that are broadly associated with both pneumonia and ILI. An additional limitation is our sample size, which is too small to permit age to be stratified into more than two groups, restricting our ability to model healthcare-seeking behaviors more precisely by smaller age groups. Finally, it is not clear whether results from one area of Guatemala with a significantly lower indigenous population than the rest of the country can be generalized to the nation as a whole.

## Conclusion

Despite the limitations of this and similar surveys, our findings indicate that Guatemala and other countries in the region can improve the estimation of the burden of influenza and other respiratory pathogens from sentinel surveillance by taking healthcare utilization into account. As a large proportion of the population with respiratory disease in Guatemala does not attend government health facilities for treatment, this approach could help correct government surveillance data for missing cases and facilitate comparison of the burden of influenza with other countries.

## Competing interests

The authors declare that they have no competing interests.

## Authors' contributions

KAL, WA, HTJ, LR and AMF conceived of the study and wrote the protocol. WA, HTJ, LR and NP supervised data collection. KAL, XZ and AJJ participated in the analysis of the study. KAL and AJJ drafted the manuscript. All authors read and approved the final manuscript.

## Pre-publication history

The pre-publication history for this paper can be accessed here:

http://www.biomedcentral.com/1471-2458/11/885/prepub
